# Large-Scale Recombinant Expression and Purification of Human Tyrosinase Suitable for Structural Studies

**DOI:** 10.1371/journal.pone.0161697

**Published:** 2016-08-23

**Authors:** Xuelei Lai, Montserrat Soler-Lopez, Harry J. Wichers, Bauke W. Dijkstra

**Affiliations:** 1 Laboratory of Biophysical Chemistry, University of Groningen, Groningen, The Netherlands; 2 ESRF-The European Synchrotron, Grenoble, France; 3 Laboratory of Food Chemistry, Wageningen University, Wageningen, The Netherlands; Karl-Franzens-Universitat Graz, AUSTRIA

## Abstract

Human tyrosinase (TYR) is a glycoprotein that initiates the first two reactions in the melanin biosynthesis pathway. Mutations in its encoding gene cause Oculocutaneous Albinism type I (OCA1), the most severe form of albinism, which is a group of autosomal recessive disorders characterized by reduced or absent production of melanin in skin, hair and eyes. Despite extensive structural and characterization studies of its homologues in lower eukaryotic organisms, the catalytic mechanism of human TYR and the molecular basis of OCA1 are largely unknown. In this work, we have carried out a large-scale recombinant expression of TYR that has enabled us to obtain high yields of pure and active protein, required for crystallization trials and screening of skin whitening agents, which is highly demanded in the cosmetic industry. Addition of an N-terminal honeybee melittin signal peptide for secretion of the produced protein into the (protein-free) medium, as well as a cleavable His-tag at the C-terminus, was crucial for increasing the yield of pure protein. We have successfully crystallized two TYR variants, in both glycosylated and deglycosylated forms, showing preliminary X-ray diffraction patterns at 3.5 Å resolution. Hence, we have established an expression and purification protocol suitable for the crystal structure determination of human TYR, which will give unique atomic insight into the nature and conformation of the residues that shape the substrate binding pocket that will ultimately lead to efficient compound design.

## Introduction

Human tyrosinase (TYR, EC: 1.14.18.1) is a key enzyme involved in the melanin biosynthesis pathway, in which it initiates the first two reactions, the hydroxylation of tyrosine to L-DOPA (L-3,4-dihydroxyphenylalanine) and the oxidation of L-DOPA to dopaquinone [1,2]. Dopaquinone then undergoes either spontaneous reactions in the presence of glutathione/cysteine to form yellow and reddish pheomelanin, or enzymatic reactions catalyzed by tyrosinase-related proteins (TYRP1 and TYRP2) to form black-brown eumelanin [3]. TYR also catalyzes the oxidation of 5,6-dihydroxyindole to indole-5,6-quinone [4]. The latter product is one of the two components necessary for the formation of eumelanin. TYR is a type I trans-membrane protein with a molecular weight of approximately 75 kDa, located inside melanosomes, which are the melanin-synthesizing organelles in melanocytes [5,6]. Its amino acid sequence contains four regions, a signal sequence (residues 1–18), an intramelanosomal domain with a binuclear copper binding site (residues 19–476), a single α-helical trans-membrane domain (residues 477–497), and a flexible C-terminal domain (residues 498–529). Moreover, TYR is a glycoprotein with seven putative N-linked glycosylation sites [7,8], which prompted the use of the enzyme as a model substrate to study the maturation of glycoproteins in the mammalian secretory pathway because of the visual nature of its enzymatic activity (melanin production) [9,10].

Mutations in the *tyr* gene cause Oculocutaneous Albinism type I (OCA1), the most severe form of albinism, which is a genetically heterogeneous group of autosomal recessive disorders characterized by reduced or absent production of melanin in skin, hair and eyes [11,12]. In humans, two different subtypes of OCA1 have been clinically identified, OCA1A and OCA1B. Mutations completely abolishing tyrosinase activity result in OCA1A, while mutations that leave some residual enzyme activity result in OCA1B, allowing accumulation of some melanin pigment over time [11]. Therefore, cases with the OCA1A type suffer from complete lifelong absence of melanin, while OCA1B patients retain the ability to tan [13]. By exploring the Human Gene Mutation Database [14], 234 pathogenic missense/nonsense mutations have been identified in OCA1 patients, affecting 169 unique amino acids, of which 53 are targeted by two or more mutations. Particularly, a subset of OCA1B mutations, including R402Q [15,16], P406L [17], and R422Q [18,19], cause the so-called temperature-sensitive oculocutaneous albinism (OCA1-TS), which leads to a temperature-sensitive form of TYR with optimal enzymatic activity at a temperature lower than 37°C; as a result, pigmentation is generally more prominent in the extremities (ears, face, and legs), where the temperature is cooler than in other parts of the body [20].

In the last decade, tyrosinases from various organisms have been extensively studied. Particularly, mushroom tyrosinase from *Agaricus bisporus* (*Ab*TYR) has been used as a model enzyme because it can be easily purified in large quantities and is commercially available. For instance, inhibitor screening for developing skin whitening agents in the cosmetic industry has been done with *Ab*TYR as a model of human TYR [21–24]. Its crystal structure was the first structure of a eukaryotic tyrosinase [25], which revealed that *Ab*TYR was the product of the *ppo3* gene and formed a tetramer of two mature tyrosinase chains and two chains of a smaller protein with a lectin-like fold. One of the copper ligands is a His residue that is covalently linked to a nearby Cys *via* a thioether bridge. More recently, several other eukaryotic tyrosinase structures have been elucidated, including *Ab*TYR encoded by the *ppo4* gene [26,27], *Aspergillus oryzae* tyrosinase [28], and *Juglans regia* tyrosinase [29,30], as well as the catechol oxidase from *Coreopsis grandiflora* [31,32]. These structures show noticeable heterogeneity in their binuclear copper active site, which has led to somewhat different proposed catalytic mechanisms. Furthermore, it is likely that human TYR may also present unique structural properties, because it does not contain a thioether bond near the active site, as found in fungal and plant tyrosinases [33], and it contains a unique cysteine-rich subdomain, the so-called EGF-domain, which is part of the intramelanosomal domain, and only present in mammalian tyrosinases and tyrosinase-related proteins, and of which the function is not known. A crystal structure of TYR might help to resolve the function of the EGF-domain, give clues on how OCA1 mutations cause albinism, and not unimportantly, may much better facilitate the design of drugs specifically targeting the human enzyme than e.g. mushroom tyrosinase.

Severe bottlenecks in the study of human TYR have been the difficulty of identifying suitable constructs for structural studies and obtaining sufficiently large amounts of protein. Very recently, both the intramelanosomal domain and full-length human TYR have been expressed as active enzymes in *Trichoplusia ni* insect larvae and in the Sf9 insect cell line, respectively [34,35]. In the former case the protein was overexpressed with a yield of ~1 mg per 10 g of larval biomass. However, the purity of the sample was not reported, and recombinant expression and extraction of proteins from insect larvae is time-consuming and requires multiple purification steps, negatively affecting the protein yield; In the latter case the protein was expressed as full-length protein including its transmembrane domain and flexible C-terminal tail, both of which would significantly increase the difficulty of obtaining well packed crystals for crystallographic studies. In the present study, aimed to produce human TYR protein for structural studies, we have established a protocol to optimize the expression yields by using the baculovirus expression vector system in *High Five* cells (derived from *Trichopulsia ni* cells). Furthermore, we have enhanced the secretion of recombinant human TYR into protein-free medium by fusing the encoding sequence with the honeybee melittin signal peptide [36]. As a result, by applying only two purification steps, nickel resin gravity-flow and ion exchange chromatography, we have been able to obtain highly pure protein samples at a final yield of ~4–6 mg per litre of culture. A main challenge of crystallizing human TYR are the intrinsically flexible N-linked carbohydrates, which are key for the enzyme’s correct folding and biological activity [37]. Thus, to enhance the success rate of crystallization, we have performed protein deglycosylation with various endoglycosidases, and, in addition, have designed a shorter construct (TYR residues 19–456) based on our crystallization results with human tyrosinase-related protein 1 (TYRP1).

## Materials and Methods

### Generation of recombinant baculovirus

The cDNA encoding full-length human TYR was synthesized by ShineGene Molecular Biotech Inc. Primers containing the honeybee melittin signal sequence and *Stu1* and *Xba1* restriction sites were synthesized by Eurofins Genomics. A first gene construct containing a *Stu1* restriction site, the honey bee melittin signal sequence, human TYR (residues 19–469), a Tobacco Etch Virus (TEV) protease cleavage site (ENLYFQG), a hexa-histidine tag and an *Xba1* restriction site, was amplified by standard polymerase chain reaction (PCR). The generated PCR product was digested with the *Stu1* and *Xba1* restriction endonucleases, purified and ligated into the pACEBac1 Baculovirus transfer vector (Geneva Biotech), previously digested with the same restriction enzymes. The resulting plasmid was confirmed by DNA sequencing (Macrogen Inc.). A second construct containing residues 19–456 was generated by applying a similar protocol. Thus, we generated two constructs, pACE-TYR (19–469) and pACE-TYR (19–456), containing an N-terminal honeybee melittin signal peptide and a C-terminal TEV-cleavable hexa-histidine tag.

The two plasmid constructs were transformed into DH10BacY bacterial competent cells (kindly provided by the EMBL Grenoble Eukaryotic Expression Facility) for the production of recombinant bacmids. The cells were incubated at 310 K overnight and subsequently plated on an agar plate containing kanamycin (50 μL/mL), gantamycin (10 μL/mL), tetracycline (10 μL/mL), isopropyl-β-D-1-thiogalactopyranoside (IPTG, 1 mM) and Bluo-Gal (100 μL/mL, Thermo Fisher Scientific) for Blue-White Screening to identify colonies containing the recombinant bacmid. Single white colonies containing recombinant bacmids were picked and inoculated into 2 mL medium for bacmid extraction and purification. Each highly pure DNA product was then mixed with DNA transfection reagent (X-tremeGEN HP, Roche) according to manufacturer’s protocol and used to transfect 3 mL of a culture containing one million of Sf21 insect cells (isolated from *Spodoptera frugiperda*). Recombinant baculovirus were harvested after 72 h and used to subsequently infect 25 mL of Sf21 cell culture. High MOI (Multiplicity Of Infection) virus stocks were harvested after around 108 h and stored at 277 K for long term use.

### Expression and purification of recombinant human TYR

For large scale expression, 2 mL recombinant baculovirus stock solution was used to infect 2 liters *High Five* cells (originally from Thermo Fisher Scientific, and kindly provided to us by the Eukaryotic Expression Facility at EMBL, Grenoble) (at a density of 0.7 million cells per mL). After culturing for 108 h, the medium was clarified by centrifugation at 6000 g and subsequently concentrated to 100 mL using a 10 kDa cut-off QuixStand Benchtop System (GE Healthcare). The concentrated medium was then incubated with 5 mL of Ni-NTA agarose resin (QIAGEN) for 20 min, which was then applied to a 20 mL gravity flow chromatography column (Econo-Pac column, Bio-Rad). The flow-through was discarded and the bound protein was washed with 2 column volumes (CV) of wash buffer (25 mM Tris-HCl, pH 7.8, 150 mM NaCl, 50 mM imidazole) and eluted with 1 CV of elution buffer (25 mM Tris-HCl, pH 7.8, 150 mM NaCl, 500 mM imidazole). TEV protease was added to the eluted protein solution and dialyzed overnight at 273 K against dialysis buffer (25 mM Tris-HCl, pH 7.8, and 50 mM NaCl) to remove the 6xHis-tag. TEV protease and uncleaved protein were depleted by a second gravity flow chromatography step. Cleaved human TYR protein in the flow-through was collected and applied to a MonoQ column (GE Healthcare) for an ion-exchange chromatography purification step, eluted with a NaCl concentration gradient from 50 to 500 mM. The pure protein eluted at approximately 150 mM NaCl. The purity of the protein was analysed by SDS-PAGE. The elution fractions containing the pure sample were finally pooled and concentrated over a 30 kDa cut-off Amicon membrane (Millipore) to ~10 mg/mL for characterization and crystallization experiments.

### Analytical gel filtration and activity assay

0.1 mL of pure human TYR sample (~10 mg/mL) was loaded onto a Superdex 200 10/300 gel filtration column (GE Healthcare) equilibrated with elution buffer (25 mM Tris-HCl, pH 7.8, 200 mM NaCl). Elution from the gel filtration column was carried out at a flow rate of 0.5 mL/min, fractionation volume of 0.3 mL, and total running time of 40 min. The single peak fractions were selected for activity assays.

TYR enzyme activity assays were performed at 298 K using 3,4-dihydroxyphenylalanine (L-DOPA) as substrate in a Nunc^™^ Edge 96-Well plate (Thermo Fisher Scientific) [38,39]. Briefly, 10 μL of 5 mM 3-methyl-2-benzothiazolinone hydrazone (MBTH) solution in water was mixed with 80 μL of L-DOPA solution (18 mM in 50 mM sodium phosphate buffer, pH 6.5). The colorimetric reaction started with the addition of 10 μL of protein solution to the reaction mixture, and was followed by monitoring the development of the pink-coloured quinone-MBTH adduct. After 5 min reaction time, an image was taken using a Photo HP Scanjet G3110 scanner (Hewlett-Packard).

### Enzymatic deglycosylation

Glycosidases PNGase F and Endo H_f_ were purchased from New England Biolabs. All enzymatic deglycosylation reactions were carried out according to the manufacturer’s manual. The efficiency of deglycosylation was evaluated by 15% SDS-PAGE under reducing conditions.

### Thermal shift assay

Protein samples were buffer exchanged (100 mM HEPES, pH 7.8, 150 mM NaCl) and diluted to 10 μM. A 50 μL solution containing 10 μL of protein sample, 10 μL of 5X *Sypro Orange* dye (Thermo Fisher Scientific) and 30 μL of water, was added in each well of a 96-well polypropylene plate. The plate was sealed with an optically clear adhesive seal (Bio-Rad) and inserted into a 3000P qPCR System (Bio-Rad). The plate was then heated from 293 to 363 K with a 1 K increment per 20 seconds. The changes in fluorescence of the *Sypro Orange* probe were recorded by the fluorescence detector of the system.

### Crystallization

Pure human TYR protein was buffer exchanged to crystallization buffer (10 mM Tris-HCl, pH 7.8, 100 mM NaCl, 0.25 mM CuCl_2_) and concentrated to 25 mg/mL. An extensive crystallization screen was performed at the HTX crystallization facility (EMBL, Grenoble) using both commercial and in-house crystallization screening solutions. All crystallization trials were set up at 293 K by sitting-drop vapor diffusion using three different protein to reservoir solution ratios (1:3, 1:1 and 3:1) in 200 nL drops. Additive screening was carried out manually at a dilution factor of 10 (HR2-138 additive screen, Hampton Research).

### X-ray diffraction analysis

The crystals were soaked in the corresponding reservoir solution supplemented with 15–25% glycerol for 5 seconds, then mounted in a cryoloop and subsequently flash-cooled in liquid nitrogen. X-ray diffraction data were collected at 100 K using a Pilatus 6M-F detector (DECTRIS) at ESRF beamline ID29 (wavelength 0.976 Å).

## Results and Discussion

### Identification of two human TYR constructs for crystallization

**Fig 1:**
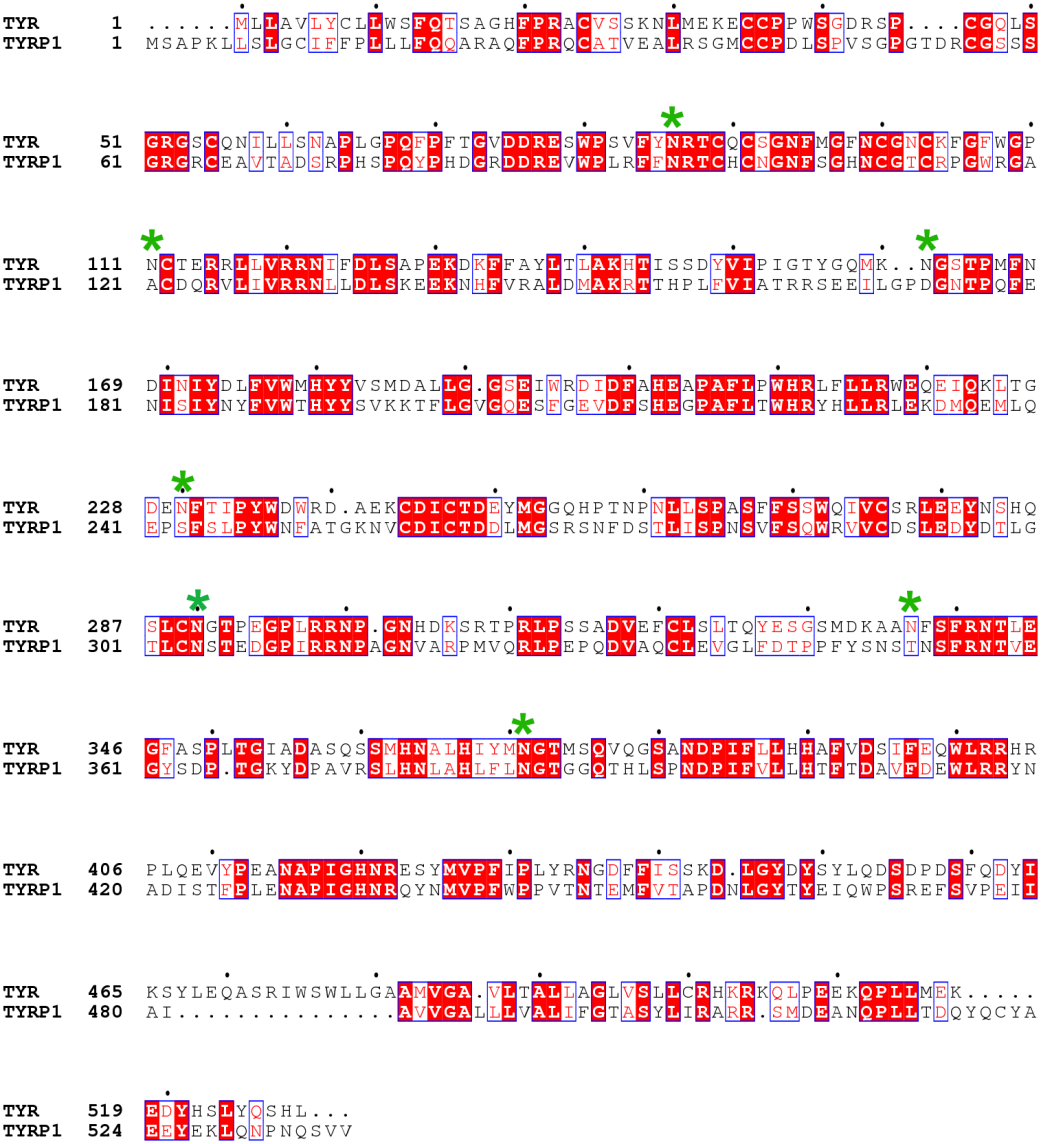
Sequence alignment of human TYR and human TYRP1. Conserved residues are highlighted in red. Putative TYR glycosylation sites are indicated with a green star. Two different TYR constructs were designed for recombinant expression, TYR456 (50 kDa, residues 19–456) and TYR469 (51.5 kDa, residues 19–469).

Mature human TYR contains three domains, the intramelanosomal domain (residues 19–476), the single trans-membrane domain (residues 477–497), and the C-terminal domain (residues 498–529). In our study, we initially selected an N-terminal construct (residues 19–469) lacking the trans-membrane and the C-terminal domains as a crystallization construct, which had been previously expressed as a soluble and active enzyme [34] (hereafter called TYR469). Furthermore, we designed an additional TYR construct spanning from residue 19 to 456 (called TYR456), since an equivalent construct of human TYRP1, which shares 43% sequence identity with human TYR (Fig 1), did give crystals. Both TYR constructs have been used in parallel in order to enhance the success rate of crystallization.

### Expression and purification of recombinant human TYR

**Fig 2:**
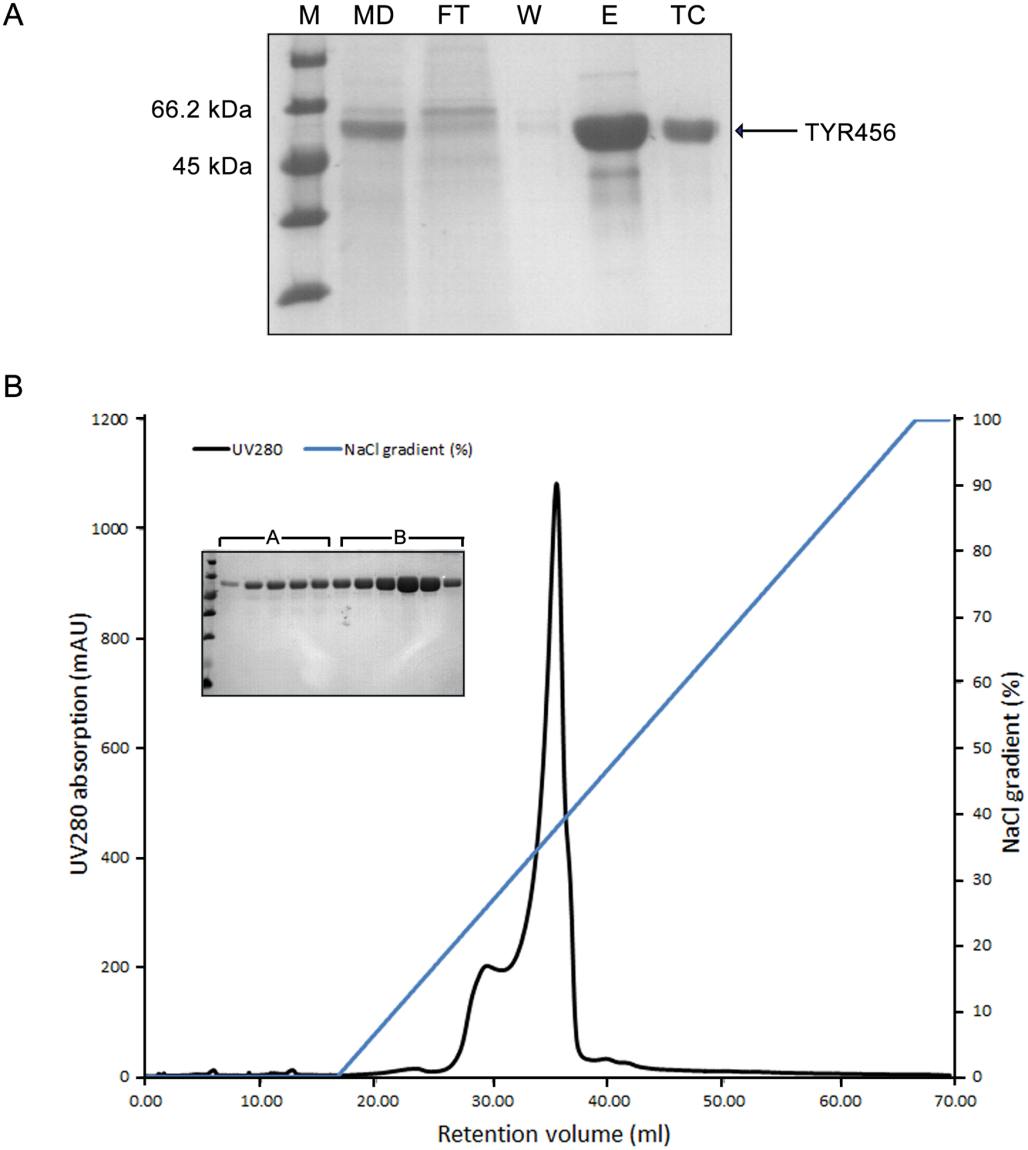
Purification of TYR456. **A.** SDS-PAGE of Ni-NTA affinity purification of recombinant TYR456 and 6xHis-tag cleavage. M, molecular weight marker; MD, concentrated medium; FT, flow-through fraction; W, wash fraction; E, elution fraction; TC, TEV-protease cleaved fraction. **B.** Ion exchange chromatogram of TYR456, upon applying a NaCl gradient from 50 mM to 500 mM. Two elution peaks are observed (A and B), both corresponding to recombinant tyrosinase samples. Peak B eluting at ~150 mM NaCl was selected for further analysis.

Both TYR456 and TYR469 were recombinantly expressed in *High Five* cells as secreted proteins in protein-free medium and purified to homogeneity. The expression yield of TYR456 was ~4–6 mg/L, two-fold higher than that of TYR469. The two variants behave similarly in solution during the purification process. A typical SDS-PAGE gel of TYR456 after the Ni-NTA resin gravity-flow chromatography step is shown in Fig 2A. The elution fractions containing human TYR were pooled for overnight 6xHis-tag TEV protease cleavage and dialysis. The majority of contaminating proteins were removed by a subsequent Ni-NTA gravity-flow chromatography step (Fig 2A, lane *TC*). An ion exchange chromatography step was applied to further increase sample purity and homogeneity. Interestingly, two peaks appeared in the elution profile, A and B, respectively (Fig 2B), although both peaks only contain TYR protein according to SDS-PAGE (Fig 2B). This is probably caused by heterogeneous glycosylation of the recombinant protein sample. Glycans from N-linked glycosylation sites may be heterogeneously processed, resulting in differences in size and surface charge of the glycoproteins. Since the fractions from peak B had the highest TYR content, they were pooled for further characterization and crystallization screenings.

### Characterization of recombinant human TYR

**Fig 3:**
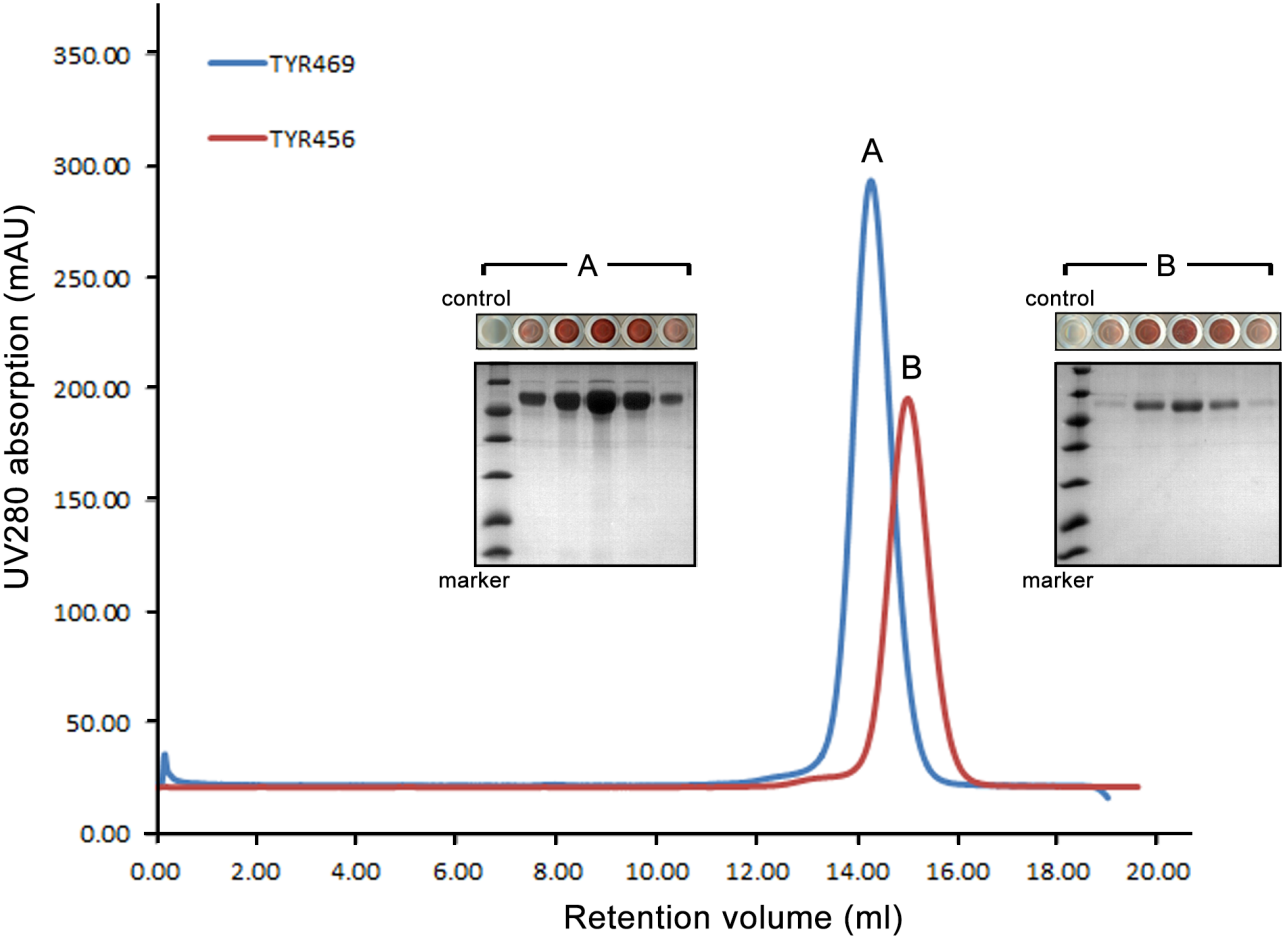
Characterization of TYR variants by gel filtration and enzyme activity assays. Overlap of the analytical gel filtration profiles of TYR456 (peak B, in red) and TYR469 (peak A, in blue) on a Superdex 200 10/300 GL column (GE Healthcare). Colorimetric activity assays with the corresponding eluted fractions using L-DOPA as substrate generated a pink or a dark pink pigment product (i.e. quinone-MBTH adduct), indicating that both variants are enzymatically active (10 μL of each elution fraction containing TYR was added in a 80 μL reaction well containing L-DOPA. 10 μL gel filtration buffer solution was used as a negative control). The apparent gradient of light pink to dark pink, and back to light pink in the reaction well indicates different protein concentrations of each elution fraction.

Stability and homogeneity of proteins in solution is crucial for crystallization. The elution profiles of an analytical gel filtration column indicate that both TYR469 and TYR456 protein constructs are monomeric in solution (Fig 3). TYR469 (Peak A) elutes slightly earlier than TYR456 (Peak B) because of its higher molecular mass. Moreover, both proteins are fully active according to the colorimetric reaction assays (Fig 3).

**Fig 4:**
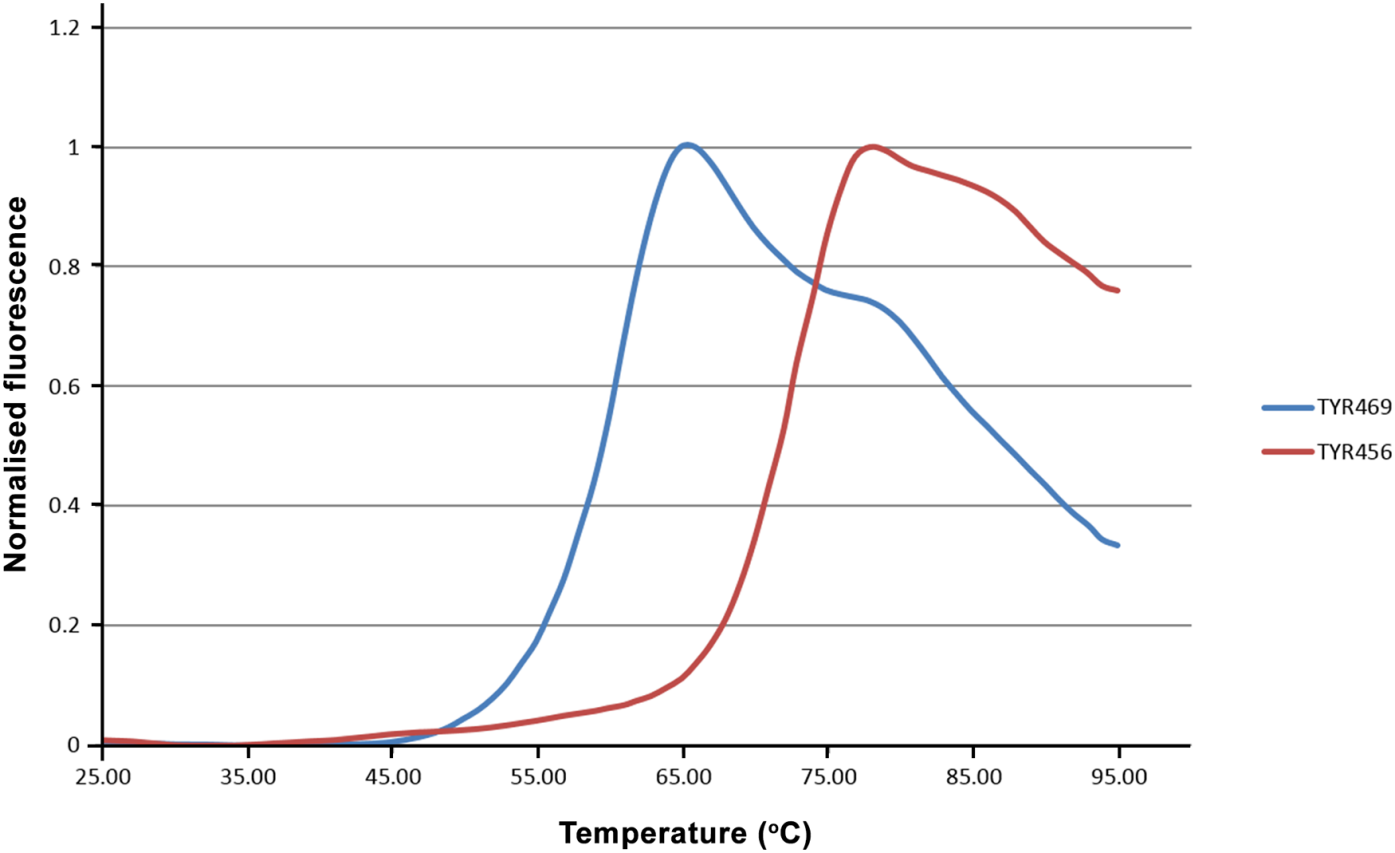
Determination of the thermal stability of the TYR variants by thermal shift assays. Fluorescence scan diagrams based on *Sypro Orange* (Thermo Fisher Scientific) binding upon thermal unfolding of TYR456 and TYR469 proteins, respectively. The apparent stability midpoint values (or melting temperatures) under the analysed buffer conditions are 72°C and 60°C for TYR456 and TYR469, respectively.

A thermal shift assay was used to evaluate the thermal stability of the recombinant proteins [40], since proteins with higher thermal stability have been shown to have a relatively higher success rate of crystallization [41]. Both TYR456 and TYR469 show reasonable thermal stability and, hence, are suitable for crystallization. TYR469 has a medium Tm (melting temperature) of 60°C, while TYR456 has a significantly higher Tm of 72°C (Fig 4). This shows that the 13 C-terminal residues of the TYR469 protein considerably destabilize the protein, the reasons for which are not known.

### Enzymatic deglycosylation of human TYR

Glycoproteins represent a challenge for crystallization due to the heterogeneity and flexibility of the oligosaccharide chains. Enzymatic deglycosylation is one of the main strategies that have been shown to enhance the success of crystallization of glycoproteins [42,43]. Nevertheless, intact glycosylated proteins have also yielded high quality crystals in some cases [44]. Therefore, both intact and deglycosylated samples were subjected to crystallization screening.

**Fig 5:**
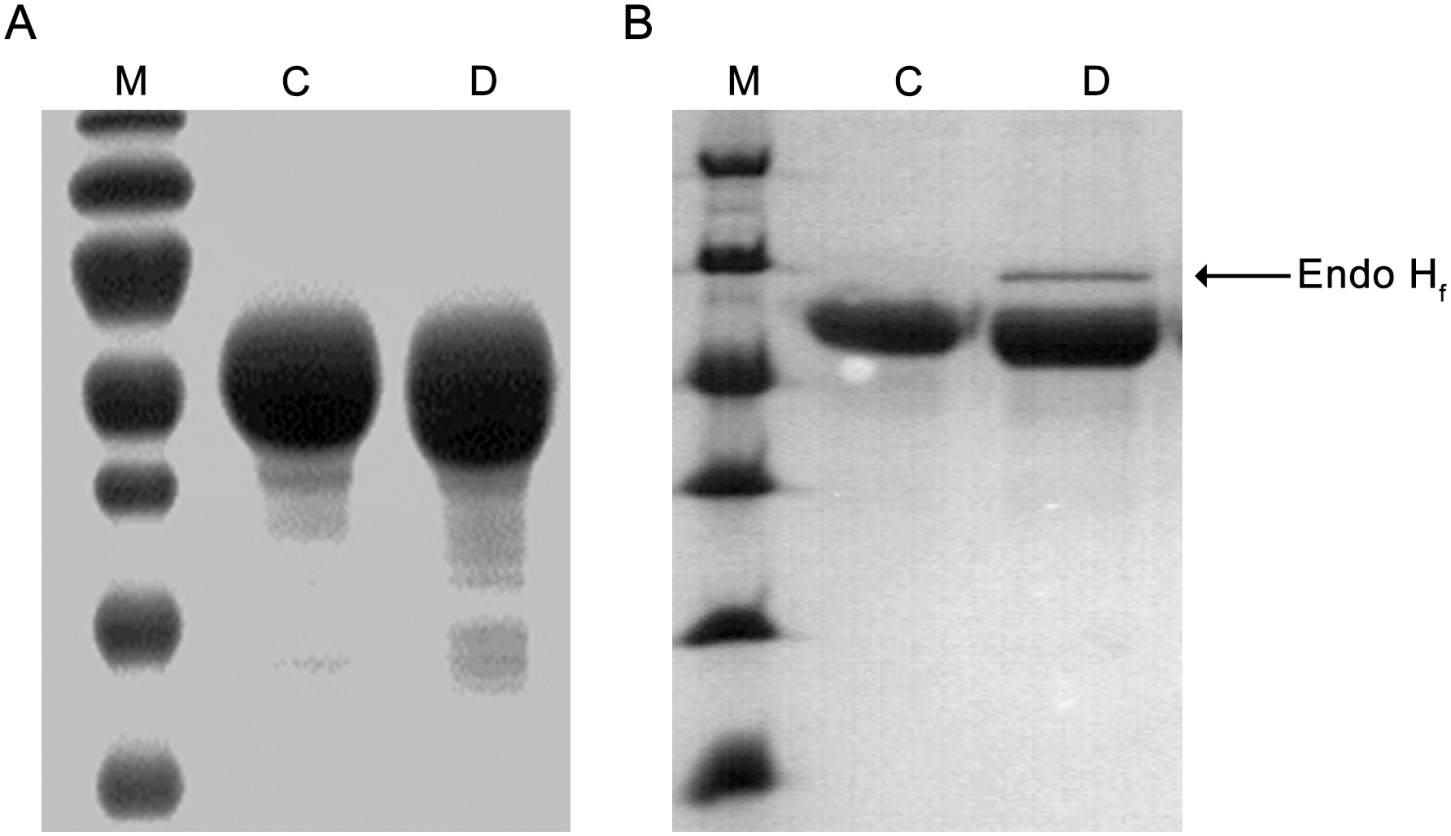
Enzymatic deglycosylation of human TYR. **A.** Deglycosylation of TYR469 with PNGase. **B.** Deglycosylation of TYR469 with Endo H_f_. M, molecular weight marker; C, non-treated protein as control; D, deglycosylated sample.

We carried out initial TYR469 deglycosylation trials with PNGase (Peptide-*N*-Glycosidase F), an amidase that cleaves between the innermost N-Acetylglucosamine (GlcNAc) and asparagine [45]. However, PNGase performed only partial deglycosylation, as evidenced by a smeared band on SDS-PAGE (Fig 5A), indicating that the treated protein sample is heterogeneous and not suitable for crystallization. This may also indicate that the innermost GlcNAc residue is fucosylated, because such fucosylation is a commonly observed modification of N-glycosylated proteins produced in insect cells [46] and PNGase is not able to cleave N-linked glycans when such a fucose is present [45]. Thus, we treated TYR469 with Endo H_f_, a recombinant fusion protein of Endoglycosidase H and maltose binding protein, which cleaves the GlcNAc-GlcNAc glycosidic bond of high mannose and some hybrid oligosaccharides from *N*-linked glycoproteins [45]. As shown in Fig 5B, Endo H_f_ deglycosylation yielded a homogeneous sample as judged by SDS-PAGE, which was then subjected to crystallization screening.

### Crystallization and preliminary X-ray diffraction of human TYR

**Fig 6:**
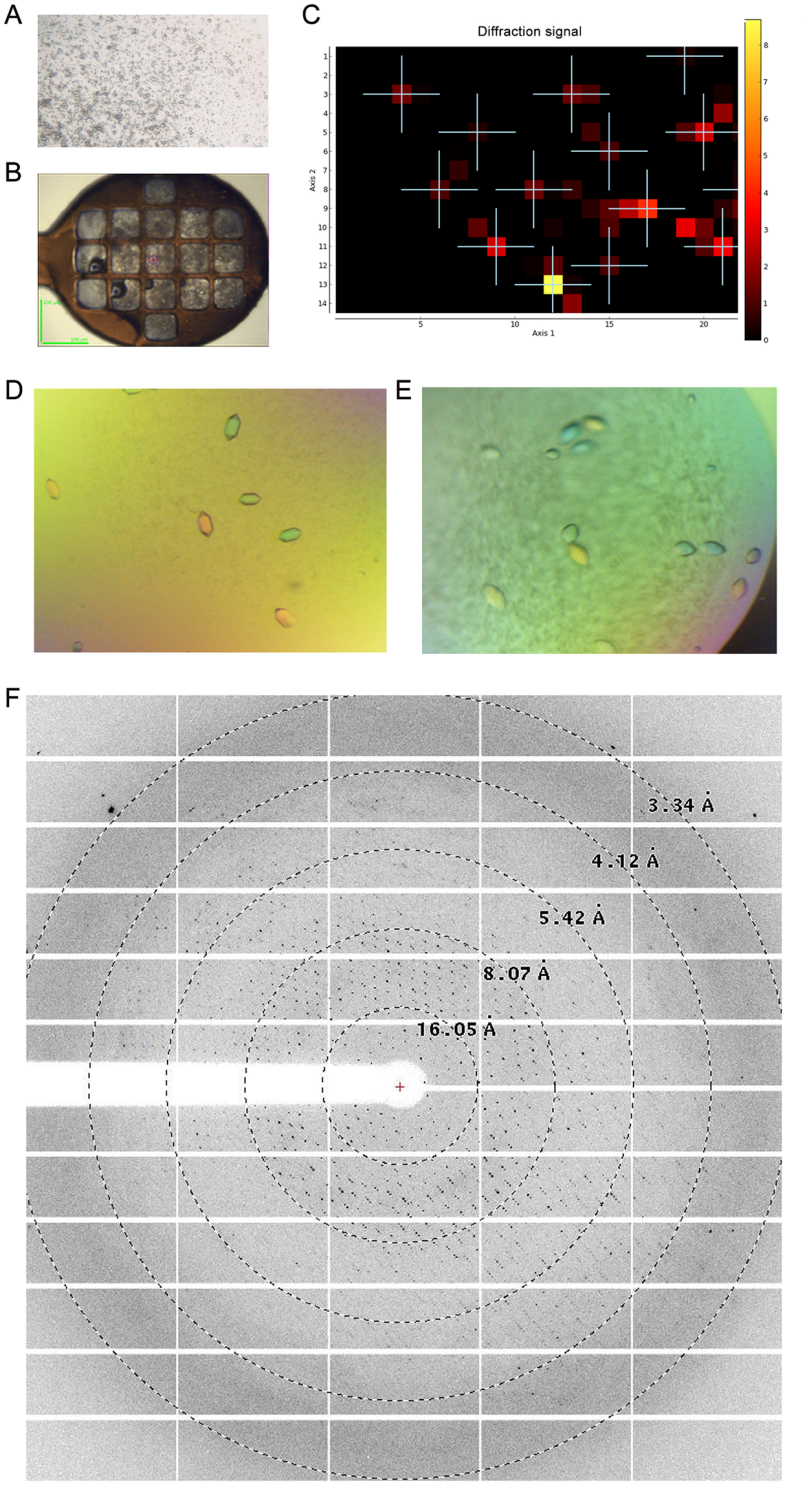
Crystallization of TYR469. **A.** Picture of the initial crystallization hit of TYR469 in 100 mM (NH)_4_SO_4_, 10 mM MgCl_2_, 50 mM MES, pH 5.9, and 18% PEG8000 (w/v). **B.** Picture of a Cryoloop containing multiple microcrystals suitable for an X-ray mesh scan. **C.** Heat map resulting from the cryoloop mesh scan. The cross spots indicate diffracting crystals positions. The quality of the diffraction is scored based on a colour gradient (from yellow—poor—to dark red—highest–). **D.** Picture of TYR469 crystals with 0.5% polyvinylpyrrolidone K15 (w/v) as an additive (HR2-138 condition E2, Hampton Research). **E.** Picture of TYR469 crystals with 10 mM spermine tetrahydrochloride as an additive (HR2-138 condition D3). **F.** Representative X-ray diffraction pattern of a crystal from E. Resolution circles are indicated.

The initial crystallization hits generated small crystals of TYR469 with a longest dimension of 5–10 μm in a reservoir solution containing 100 mM (NH)_4_SO_4_, 10 mM MgCl_2_, 50 mM MES pH 5.9, and 18% PEG8000 (w/v) (Fig 6A). Since the crystals were too small for being individually mounted in a cryoloop for diffraction tests, we applied the newly developed *MeshAndCollect* multi-crystal data collection approach at ESRF beamline ID23-1 [47]. TYR469 protein X-ray diffraction was confirmed (Fig 6B and 6C). Extensive optimization screens were set up with no improvement in diffraction quality. Finally, an additive screen generated much larger crystals, with a longest dimensions of 100 μm as shown in Fig 6D and 6E. More than 300 crystals were tested for diffraction. Some of them, optimized by adding 10 mM spermine tetrahydrochloride to the crystallization drop, had improved diffraction up to 3.5 Å.

**Fig 7:**
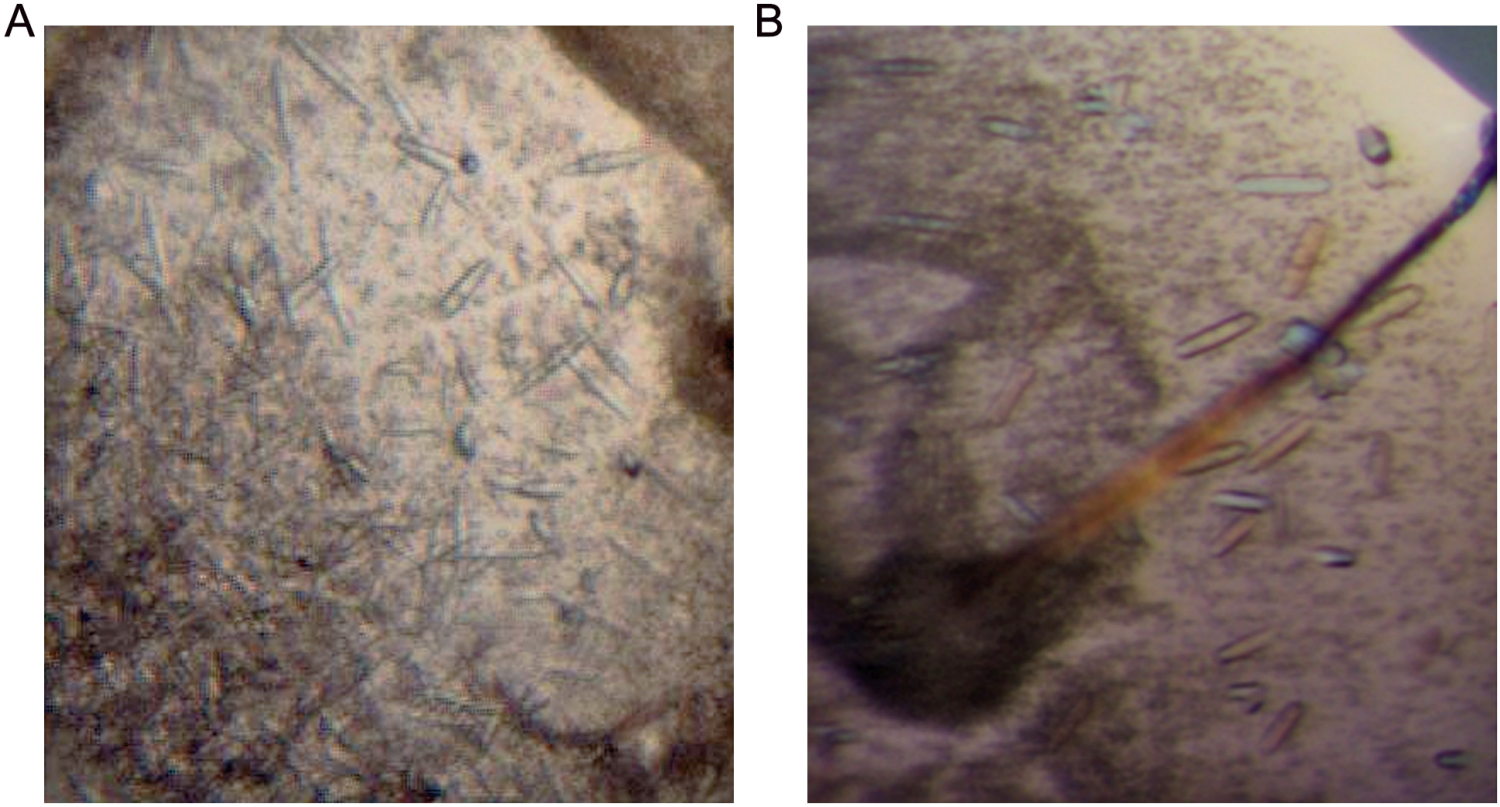
Crystallization of TYR456. **A.** Initial crystallization hit of TYR456 in 0.1 M HEPES buffer, pH 7.5, 20% w/v polyethylene glycol 10000. **B.** Optimized crystals in 0.1 M HEPES buffer, pH 7.5, 10% w/v polyethylene glycol 10,000 and 10% w/v polyethylene glycol 20,000.

In the meantime, the intact TYR456 protein (without enzymatic deglycosylation) was subjected to crystallization screening. Rod-like crystals were generated in a condition of 0.1 M HEPES, pH 7.5, 20% PEG 10000 (w/v) (Fig 7). Crystals were further optimized by mixing PEG10000 and PEG20000 (Fig 7). Crystals with a defined external shape and reasonable size were obtained but showed poor diffraction up to ~6 Å only. Despite the high concentration of tyrosinase in the crystallization set-ups, no browning of the protein due to tyrosinase activity was observed.

## Conclusions and Perspectives

Human TYR initiates the first two reactions in the melanin biosynthesis pathway. It shares ~40% sequence identity and ~70% similarity with TYRP1 and TYRP2. Thus, a high resolution crystal structure of human TYR is not only essential to understand the catalytic mechanism and the albinism-associated mutations of its own, but may also shed light on the underlying mechanisms of TYRP1 and TYRP2 at the atomic level. Moreover, novel skin whitening agents targeting human TYR are highly demanded in the cosmetic industry [48–50]. Hence, a crystal structure of human TYR in complex with bound substrates/inhibitors would give unique atomic insight into the nature and conformation of the residues that shape the substrate binding pocket, which is essential for efficient compound design.

One of the major challenges in crystal structure determination of TYR is the obtention of highly pure and active protein in sufficient amounts. In the last two decades, several independent groups have set up recombinant expression protocols for producing TYR using different expression systems. Kong and colleagues reported the first expression tests of TYR using a bacterial expression system. They were able to obtain in good yield both full-length protein [51] and the intramelanosomal domain [52], with good tyrosinase activity. In contrast, Chen and colleagues expressed TYR in the same system, but only obtained insoluble protein, from which active protein was obtained by refolding in a detergent-containing buffer [53]. However, these protocols were never reproduced by other groups [35], and as expected from expression in a prokaryote, the obtained protein was not glycosylated, which had been shown to be important for activity and solubility of TYR [37]. Thus, to tackle the reproducibility and glycosylation problem, Dolinska and colleagues produced the intramelanosomal domain of TYR with a fused heterologous signal peptide in a fully active and glycosylated form, using *Trichoplusia ni* larvae as expression host [34]. Although the protein solubility and overexpression yield was improved, recombinant expression in insect larva is time-consuming and, thus, not ideal for structural studies which requires long-term provision of the protein sample for screening many crystallization conditions. Furthermore, the reported purification protocol required multiple steps, which are likely to cause loss of protein. More recently, Fogal and colleagues overexpressed active, full-length TYR in *Spodoptera frugiperda* (sf9) cells using a baculovirus expression system [35]. Yet, the full-length sample contains the single α-helical trans-membrane domain, and the flexible C-terminal domain, both of which are likely detrimental for crystallization. In this study, we set up an easy protocol to overproduce active TYR, in which only two purification steps are needed to obtain highly pure protein sample. The protocol provides a consistent high expression yield of 4–6 mg per litre of culture, which is not only useful for crystallization screening, but also can be applied to carry out high-throughput screening for skin whitening agents.

Another major challenge in crystallizing TYR is likely the presence of N-linked carbohydrate residues due to their intrinsic flexibility and heterogeneity. Indeed, glycoproteins are troublesome for crystallization in general, as evidenced by the dearth of human glycoproteins in the Protein Data Bank [54]. In this study, we developed a deglycosylation protocol suitable for crystallization purposes for TYR. This allowed us to obtain good TYR crystals that show diffraction data to near-atomic resolution of 3.5 Å in our preliminary diffraction tests. Future perspectives include increasing the diffraction limit of the crystals. Many strategies are available for this purpose. For example, crystal optimization including seeding [55,56] and additive screening [57], and crystal post-manipulation including dehydration [58,59] and annealing [60] have all shown to be successful for several proteins.
